# Clinical Practice Guidelines for the Diagnosis of Inflammatory Bowel Disease (IBD): A Scoping Review

**DOI:** 10.1002/hsr2.71382

**Published:** 2025-10-14

**Authors:** Chelsea Doyle, Charlotte Combes, Meri Lioulios, Mara Koutsouridis, Shannon Leyshon, Elio Arruzza

**Affiliations:** ^1^ UniSA Allied Health & Human Performance University of South Australia South Australia Australia

**Keywords:** colitis inflammatory bowel disease ulcerative, Crohn disease, practice guideline

## Abstract

**Background and Aims:**

Inflammatory bowel disease (IBD) is a chronic autoimmune disorder. However, inconsistencies in diagnostic processes exist. This scoping review aimed to evaluate current global clinical practice guidelines (CPGs) to assist clinical decision‐making.

**Methods:**

The Preferred Reporting Items for Systematic Reviews and Meta‐Analyses extension for Scoping Reviews (PRISMA‐Scr) was utilised. CPGs were identified by searching electronic databases Medline, Scopus, Embase and grey literature until March 2024. Included guidelines were published within the last 5 years in English, with recommendations for diagnosis of IBD. Publications were independently extracted and critically appraised by two reviewers using the AGREE‐II tool. Diagnostic recommendations were extracted and categorized.

**Results:**

Eleven CPGs met the inclusion criteria of this review. A total of 21 recommendations were identified and classified in relation to medical history and physical examination, blood testing, computed tomography (CT), sonography, endoscopy, colonoscopy, and magnetic resonance imaging (MRI).

**Conclusions:**

Diagnostic recommendations varied across CPGs, reflecting differences in regional practices and methodological rigor. The methodological quality was inconsistent, particularly regarding the applicability of recommendations. The findings highlight the need for harmonization of diagnostic criteria, improved guideline development processes, and further research to address gaps in clinical guidance and implementation.

## 1 Introduction

Inflammatory bowel disease (IBD) is a chronic autoimmune disorder which describes inflammation of the gastrointestinal (GI) tract; the two main diseases being Crohn's disease (CD) and ulcerative colitis (UC) [1]. UC is defined as inflammation limited to the large intestine, while CD can occur anywhere along the GI tract [2]. Along with location, there are variations in type and pattern of inflammation, and gut wall thickness involved [2]. These conditions are generally not associated with increased mortality, however, lead to significant morbidity and decreased quality of life [1].

Whilst IBD has a pronounced impact globally, the epidemiology and aetiology of IBD is vastly unknown. IBD diagnosis follows a bimodal distribution, with first peak of incidence between 15 and 25 years‐of‐age, and a second peak during the fifth to seventh decades [3]. Alatab et al. [4] report a total of 6.9 million cases globally, with 3.9 million cases being females. Historically, IBD was associated with Western countries, with the United States of America (USA) accumulating nearly a quarter of diagnoses. However, recent studies have determined a more exponential growth of IBD patients within developing countries. Ng et al. [5] identified a dramatic increase in age‐standardised prevalence rate from 1990 to 2017, specifically in regions that formerly had low prevalence, including East and South Asia, Oceania, and sub‐Saharan Africa.

Clinical practice guidelines (CPGs) aim to provide current medical recommendations to ensure patients globally receive appropriate care [6]. Several national and international CPGs relating to the diagnosis of IBD have been published. However, there is variable diagnostic recommendations, potentially owing to inconsistencies present across these guidelines. Furthermore, advances in imaging technology and diagnostic techniques have provided more options for treating clinicians. In a study comparing four CPGs, Okobi et al. [7] noted diagnostic methods of all sources demonstrated variation. While all CPGs utilised endoscopy as a primary diagnostic test, some sources also included imaging such as computed tomography (CT), magnetic resonance imaging (MRI), and colonoscopy, as well as biopsy. By reviewing key recommendations specific to diagnosis in the context of CPGs for IBD patients, we aimed to synthesise and appraise the current evidence, whilst identifying trends and deficiencies where future research is required.

## 2 Methods

This scoping review utilised the “Preferred Reporting Items for Systematic Reviews and Meta Analyses extension for Scoping Review” (PRISMA‐ScR) statement [8]. Written informed consent and ethical approval were not required because of the nature of the study.

### 2.1 Research Question

The research question was guided by the PCC framework (Population, Context, and Concept) [9]. The following question guided this study: What are the key recommendations from clinical practice guidelines regarding diagnosis of patients with inflammatory bowel disease?

### 2.2 Inclusion and Exclusion Criteria

This review included CPGs and guidance documents with recommendations for the diagnosis or management of IBD. We included institutional, national, and international documents. Guidelines were excluded if they were (i) not published in English, (ii) primary studies, (iii) secondary studies (other than CPGs), (iv) withdrawn, and (v) focussing on paediatrics specifically. A 5‐year date range was applied to the search, and when more than document was produced by the same author, the most recently issue was considered.

### 2.3 Study Retrieval and Selection

To identify relevant sources, the electronic databases Medline, Scopus and Embase were searched until March 2024. Additionally, grey literature databases UpToDate, Guidelines International Network (GIN), the National Institute for Health and Care Excellence, and the Agency for Health Care Research and Quality were searched. Medline and Embase were searched using MeSH headings, where relevant.

**Figure 1:**
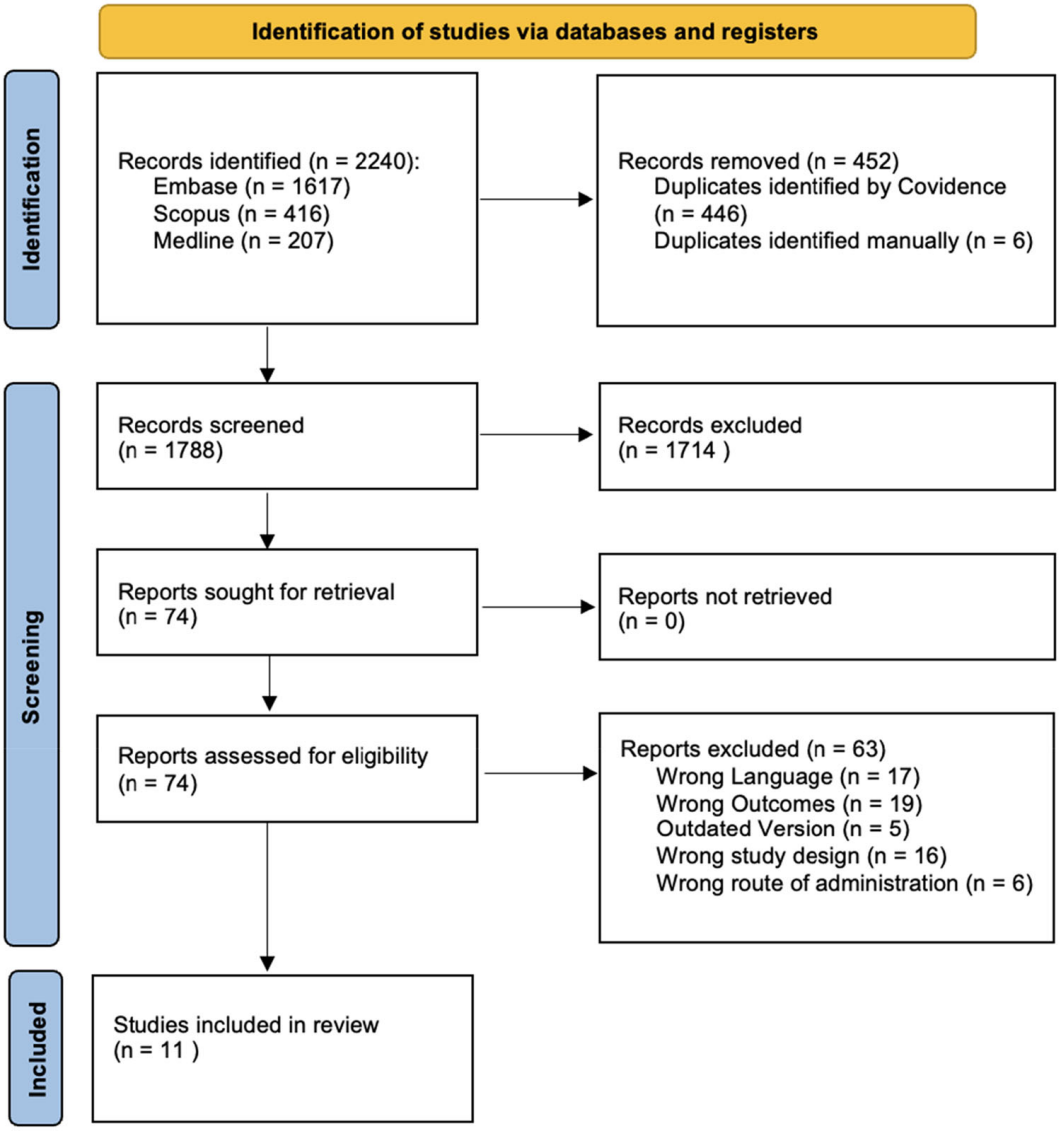
PRISMA diagram.

Using Covidence software (Melbourne, Australia), two authors (MK and SL) screened the titles and abstracts of the studies with reference to the eligibility criteria. Conflicts over eligibility criteria were resolved by a third author (ML). This process was repeated for full text screening to determine the inclusion of guidelines in our review. The screening process was summarised within a PRISMA flow diagram (Figure 1), which outlines the studies origin and reasons for exclusions.

### 2.4 Data Charting and Items

After initial screening of the CPG's, two review authors (CD, CC) independently extracted data from the included sources. Date extraction tables were then designed to facilitate extraction of the following elements: (1) general study characteristics (title, country of origin, year of publication, organisation), (2) aims of the CPG, (3) target population, and (4) key recommendations for the diagnosis of IBD in clinical practice. Tables were accompanied by a narrative synthesis.

### 2.5 Critical Appraisal

Critical appraisal using the AGREE‐II instrument was implemented. This consisted of 23 questions across six domains: scope and purpose, stakeholder involvement, rigour of development, clarity of presentation, applicability, and editorial independence which were used to assess each guideline. Each question was rated on a scale of one to seven, with seven indicating high methodological quality. Guidelines were deemed satisfactory if they achieved a score of at least 50% in each domain [10].   Two reviewers (ML and MK) assessed the methodological quality of the guidelines using the AGREE‐II instrument. Any discrepancies were resolved through discussion, with a third reviewer (CC) consulted if necessary. AGREE‐II scores were calculated as a percentage by adding up the scores and dividing by the maximum possible score (ranging from 0% to 100%) for each appraiser and were presented in a graph.

## 3 Results

### 3.1 Study Selection

A total of 2240 articles were identified when imported into Covidence. 452 articles were removed as duplicates, yielding 1788 studies to be screened. After title and abstract screening, 74 studies then underwent full‐text screening. Following final full‐text screening, 11 guidelines satisfied the inclusion criteria. Figure 1 displays the PRISMA flow diagram summarising this process.

### 3.2 General Characteristics

General characteristics of the 11 CPGs are displayed in Table 1. It highlights the range of CPGs collected on a global scale, specifically the United States (*n* = 2), Poland (*n* = 2), Germany, India, United Arab Emirates (UAE), Japan, Saudi Arabia, Russia and Europe collectively (*n* = 1). The CPGs share the common aim to consolidate and develop a recommendation for health professionals in aiding the diagnosis of IBD for their respective countries/region; some CPGs had a focus on either UC (*n* = 3) or CD (*n* = 4).

**Table 1:** The characteristics of each included CPG, detailing their aims and content.

CPG number	Citation	Country	Organisation	Aim of the CPG	UC	CD
1	Maaser et al. [11]	EUR	European Crohn's and Colitis Organisation (ECCO)/European Society of Gastrointestinal and Abdominal Radiology (ESGAR)	Merge and update the former ECCO‐ESGAR Imaging Guideline and the former ECCO Endoscopy Guideline for IBD.	✓	✓
2	Kucharzik et al. [12]	DEU	German Society for Gastroenterology and Metabolic Diseases	Develop a guideline for the treatment of UC which is easily incorporated in all types of medical practices.	✓	
3	Kedia et al. [13]	IND	Indian Society of Gastroenterology (ISG)/Indian Radiological and Imaging Association (IRIA)	Develop a guideline for the diagnosis and management of IBD to be used as a reference in a clinical, teaching and research setting.		✓
4	Rubin et al. [14]	USA	American College of Gastroenterology	Develop a guideline which highlights the preferred method of diagnosis and management of adults with UC in America.	✓	
5	Kim et al. [15]	USA	American College of Radiology	Consolidate the use of diagnostic imaging for the initial diagnosis of CD.		✓
6	Alkhatry et al. [16]	UAE	Emirates Society of Gastroenterology	Consolidate the diagnosis of IBD, through endoscopic, histological, radiological, and/or biochemical results.	✓	✓
7	Nakase et al. [17]	JAP	Japanese Society of Gastroenterology	Support decision‐making in clinical practice by providing information on the diagnosis, treatment, and follow‐up of IBD.	✓	✓
8	Łodyga et al. [18]	POL	Polish Society of Gastroenterology	Provide therapeutic and diagnostic technique recommendations for adults with CD.		✓
9	Mosli et al. [19]	SAU	Saudi Gastroenterology Association (SGA)/Saudi Society of Clinical Pharmacology (SCCP)	Provide recommendations for the diagnosis and treatment of IBD in adult patients.	✓	✓
10	Eder et al. [20]	POL	Polish Society of Gastroenterology	Provide recommendations for the diagnosis and treatment of UC in adult patients.	✓	
11	Shelygin et al. [21]	RUS	Multiple	Provide recommendations for the diagnosis, treatment, and management for CD.		✓

### 3.3 Key Findings

Table 2 summarises the key recommendations from the 11 included CPGs. Evaluation of patient past medical history was recommended in all CPGs (100%). Five (45.4%) CPGs recommend performing contrast‐enhanced CT, and three (27.2%) CPGs suggested considering CT only if MRI or US is unavailable. Six (54.5%) CPGs recommend using US in conjunction with other imaging modalities to diagnose IBD. Endoscopic procedures were recommended by nine (81.8%) CPGs, especially esophagogastroduodenoscopy for patients with upper GI symptoms, as recommended by seven (63.6%) CPGs. Capsule endoscopy was recommended by four (36.3%) CPGs, whilst Ileo‐colonoscopy with a minimum of two mucosal biopsies from inflamed and uninflamed sites was recommended by eight (72.7%) CPGs. Sigmoidoscopy was recommended for UC diagnosis by four (36.3%) CPGs. MRI was recommended to complement endoscopic results in specific cases by seven (63.6%) CPGs, with two (18.2%) CPGs suggesting MRI is somewhat recommended for suspected perianal involvement.

**Table 2:** Summary of recommendations regarding IBD diagnosis of included CPGs.

Recommendation	Specific CPGs featuring recommendation	Total CPGs featuring recommendation
*Assessment of patient medical history and physical examination:*		
1. Assessment of patient medical history before diagnostic tests/imaging.	1, 2, 3, 4, 5, 6, 7, 8, 9, 10, 11	11
2. There is no global reference for the diagnosis of IBD and is rather based on a multitude of diagnostic procedures.	1, 2, 3, 4, 5, 6, 7, 8, 9, 10, 11	11
*Blood tests:*		
1. Blood workup to determine the levels of C‐reactive protein, iron, liver enzymes, protein, and blood/platelet counts.	2, 6, 11	3
2. CD cannot be ruled out by unremarkable blood test results.	8	1
*Computed tomography:*		
1. CT abdomen‐pelvis is an integral component of IBD diagnosis.	2, 4, 5, 7, 8, 9, 11	7
2. CT with contrast provides evidence of inflammation of an affected gastrointestinal segment and evaluation of CD complications, including bowel obstruction, fistula formation, and abscess formation.	4, 5, 7, 9, 11	5
3. Only be used if MRI or US is unavailable.	1, 3, 6	3
*Sonography:*		
1. Used in conjunction with other imaging modalities for conclusive diagnosis.	2, 3, 5, 6, 8, 11	6
2. Abdominal US for visualisation of the small intestine in patients with IBD.	1, 2, 3	3
3. Not a necessity due to accessibility issues involved; e.g. staff, facilities, and expertise.	3	1
4. Improved visualisation with use of oral contrast.	5, 6	2
*Endoscopy:*		
1. Endoscopic procedures are essential for the diagnosis/localisation of IBD.	1, 2, 3, 4, 7, 8, 9, 10, 11	9
2. Esophagogastroduodenoscopy should be performed for patients presenting with symptoms originating from the upper gastrointestinal tract.	1, 3, 4, 7, 8, 9, 11	7
3. Capsule endoscopy should be considered.	1, 8, 9, 11	4
4. Mucosal biopsies of focal gastritis from esophagogastroduodenoscopy to support diagnosis of CD only.	6, 9	2
*Colonoscopy:*		
1. Ileo‐colonoscopy with minimum of two mucosal biopsies from inflamed and uninflamed sites around the colon and ileum.	1, 2, 4, 6, 8, 9, 10, 11	8
2. Sigmoidoscopy should be considered if symptoms suggest UC.	1, 6, 10, 11	4
3. Colonoscopy to measure extent of disease rather than as a diagnostic tool.	1, 3	2
4. Colonoscopy necessary to confirm the diagnosis if UC is suspected.	7	1
*Magnetic resonance imaging:*		
1. Used to compliment endoscopy and if colitis is not clearly identified and/or to differentiate between UC and CD and/or if patient is allergic to contrast.	2, 3, 4, 5, 6, 9, 10	7
2. If there is suspected perianal involvement MRI of the pelvis and perineum, should be the first line of investigation.	3, 11	2

### 3.4 Critical Appraisal

**Figure 2:**
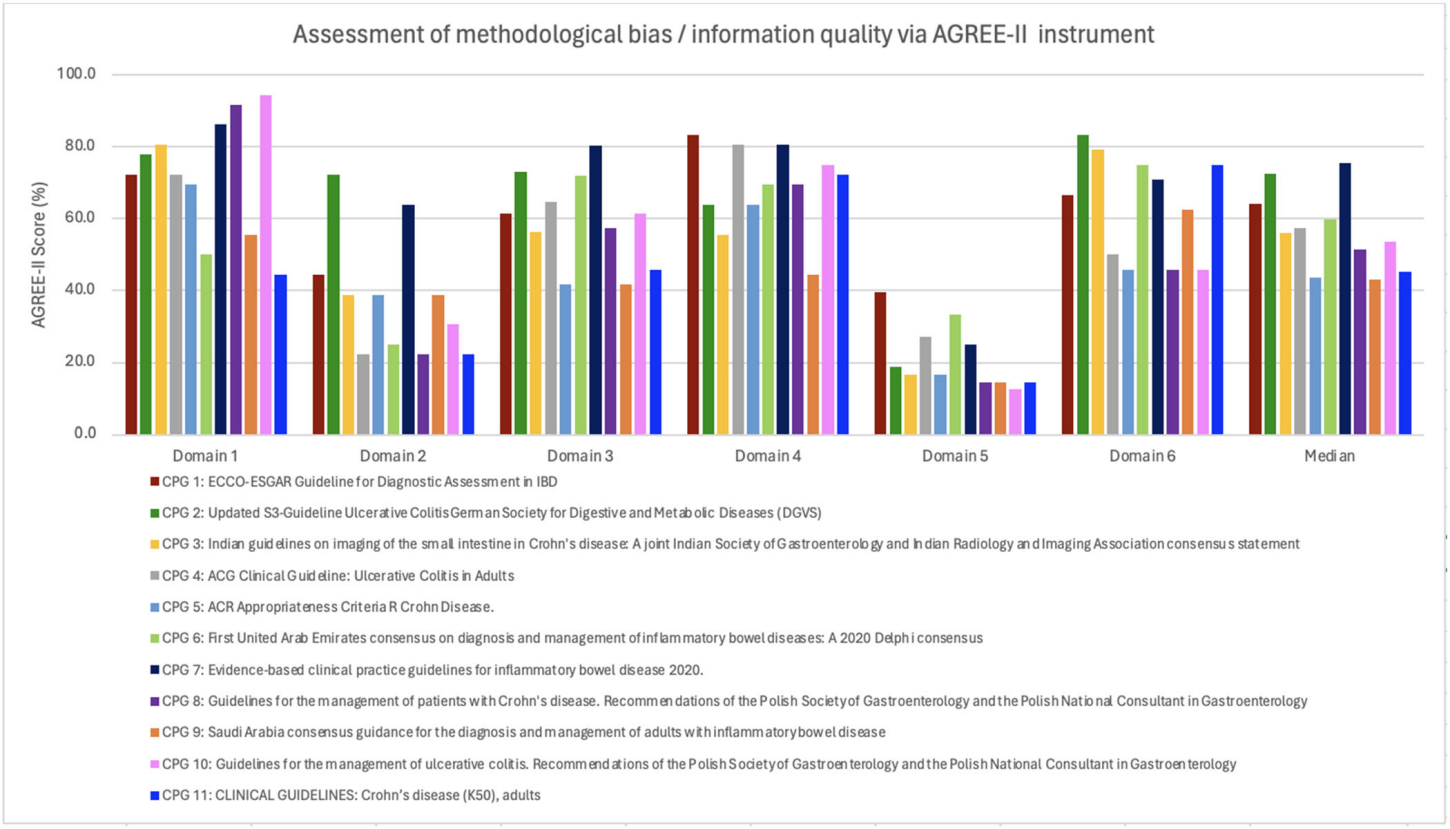
Assessment of methodological bias concerning six domains of the AGREE‐II tool for the 11 CPGs. CPGs, clinical practice guidelines.

Figure 2 displays the methodological quality of each included CPG as determined using the AGREE‐II instrument. The overall mean AGREE‐II scores for all the studies across the six domains (scope and purpose, stakeholder involvement, rigor of development, clarity of presentation, applicability, and editorial independence) were 72.2%, 38.1%, 59.6%, 68.9%, 21.2% and 63.6% respectively. The overall guideline quality, with a maximum score of seven, ranged between a score of three and six.

Domains one (scope and purpose) and four (clarity of presentation) were most successfully performed. These domains assess the capacity for the audience to clearly identify information relating to IBD diagnosis, and the language and format of the guideline, respectively. Contrastingly, domain five (applicability) held the lowest scores. This represents a lack of discussion relating to application barriers, inclusive of cost and resource implications.

## 4 Discussion

In this review, we identified 21 key recommendations derived from 11 CPGs that addressed diagnostic methods for IBD. The guidelines included in this review support an increased effort to evaluate and refine the diagnostic management for patients with IBD, as indicated by the aim of each source.

The diagnosis of IBD can be challenging. This is reinforced by the identified variations in diagnostic methods across included CPGs, consistent with previous literature noting inconsistencies in diagnostic processes and their potential impact on patient care [7, 13]. By considering the number of guideline documents that support a particular recommendation, in conjunction with the recentness and quality of that guideline document, it is hoped clinicians can use the results of our review to inform their clinical decisions. The presence of certain diagnostic recommendations in a minimal number of guidelines may reflect regional differences in healthcare infrastructure, resource availability, or population needs. For example, some guidelines may emphasize a particular imaging modality based on local access to equipment and funding, while others may omit them due to limited feasibility in low‐resource settings. Additionally, variation in methodology, such as the strength of evidence considered or the composition of guideline panels, may influence which recommendations are prioritized. Conversely, the diagnostic recommendations consistently included across multiple guidelines likely reflect areas of strong clinical consensus and high‐quality supporting evidence. These recommendations are well‐established in clinical guidelines and practice, and supported by international studies and expert agreement.

To support harmonization of diagnostic criteria across CPGs, collaboration between institutions and societies could be fostered through joint consensus panels or working groups, incorporating representation from low‐ and middle‐income settings. Furthermore, to improve underperforming domains such as applicability in these guidelines, future guideline development should explicitly address implementation strategies, cost considerations, and infrastructure needs. Inclusion of multidisciplinary stakeholders, including patients, could also enhance the relevance and feasibility of guidelines in real‐world settings.

This review of CPGs incorporated strategies to minimise bias, including a thorough screening/selection process, and the use of multiple reviewers to evaluate methodological quality and perform data extraction/synthesis. Nonetheless, the review was limited to guidelines published in English and within the last 5 years, potentially omitting high‐quality guidelines containing valuable recommendations for IBD diagnosis. Additionally, the use of the AGREE‐II tool might have introduced inherent bias, as the scoring of the included CPGs was dependent on the subjective assessments of the reviewers.  These limitations should be considered when interpreting the findings. However, despite these constraints, this review provides valuable insights into current global practices and offers a foundation for improving consistency in IBD diagnosis.

## 5 Conclusion

This review has evaluated the quality of current evidence and recommendations from CPGs. Twenty‐one key recommendations derived from 11 CPGs synthesised from multiple sources internationally. The AGREE‐II tool was used to assess the methodological quality of the CPGs in which these recommendations were drawn from, and found varying levels of performance across six domains. It is hoped this review will allow for clinicians to consider these recommendations and apply this knowledge with consideration of their local contexts.

## Author Contributions

**Chelsea Doyle:** investigation, writing – original draft. **Charlotte Combes:** investigation, investigation, writing – original draft, writing – original draft. **Meri Lioulios:** investigation, writing – original draft. **Mara Koutsouridis:** investigation, writing – original draft. **Shannon Leyshon:** investigation, writing – original draft. **Elio Arruzza:** conceptualization, methodology, resources, supervision, writing – original draft; writing – review and editing.

## Conflicts of Interest

The authors declare no conflicts of interest.

## Transparency Statement

The corresponding author Elio Arruzza affirms that this manuscript is an honest, accurate, and transparent account of the study being reported; that no important aspects of the study have been omitted; and that any discrepancies from the study as planned (and, if relevant, registered) have been explained. All authors have read and approved the final version of the manuscript. All authors had full access to all of the data in this study and takes complete responsibility for the integrity of the data and the accuracy of the data analysis.All authors affirm that this manuscript is an honest, accurate, and transparent account of the study being reported; that no important aspects of the study have been omitted; and that any discrepancies from the study as planned (and, if relevant, registered) have been explained.

## Data Availability

Due to the secondary nature of this study, the data reported in this manuscript have been previously published. Findings from the data extraction stage have been reported in the included studies.

## References

[1] S. M. Borowitz , “The Epidemiology of Inflammatory Bowel Disease: Clues to Pathogenesis?,” Frontiers in Pediatrics 10, no. 1 (2023): 1103713. 36733765 10.3389/fped.2022.1103713 PMC9886670

[2] Gastroenterological Society of Australia (GESA) . Inflammatory Bowel Disease Clinical Update. (2018). https://www.gesa.org.au/public/13/files/Education%20%26%20Resources/Clinical%20Practice%20Resources/IBD/2018_IBD_Clinical_Update_May_update.pdf.

[3] E. V. Loftus and W. J. Sandborn , “Epidemiology of Inflammatory Bowel Disease,” Gastroenterology Clinics of North America 31, no. 1 (2002): 1–20. 12122726 10.1016/s0889-8553(01)00002-4

[4] S. Alatab , S. G. Sepanlou , K. Ikuta , et al., “The Global, Regional, and National Burden of Inflammatory Bowel Disease in 195 Countries and Territories, 1990–2017: A Systematic Analysis for the Global Burden of Disease Study 2017,” Lancet Gastroenterology & Hepatology 5, no. 1 (2020): 17–30. 31648971 10.1016/S2468-1253(19)30333-4 PMC7026709

[5] S. C. Ng , H. Y. Shi , N. Hamidi , et al., “Worldwide Incidence and Prevalence of Inflammatory Bowel Disease in the 21st Century: A Systematic Review of Population‐Based Studies,” Lancet 390, no. 10114 (2017): 2769–2778. 29050646 10.1016/S0140-6736(17)32448-0

[6] M. H. Murad , “Clinical Practice Guidelines,” Mayo Clinic Proceedings 92, no. 3 (2017): 423–433. 28259229 10.1016/j.mayocp.2017.01.001

[7] O. E. Okobi , I. O. Udoete , O. O. Fasehun , et al., “A Review of Four Practice Guidelines of Inflammatory Bowel Disease,” Cureus 13, no. 8 (2021): e16859. 34513436 10.7759/cureus.16859 PMC8413108

[8] A. C. Tricco , E. Lillie , W. Zarin , et al., “PRISMA Extension for Scoping Reviews (PRISMA‐ScR): Checklist and Explanation,” Annals of Internal Medicine 169, no. 7 (2018): 467–473. 30178033 10.7326/M18-0850

[9] D. Pollock , M. D. J. Peters , H. Khalil , et al., “Recommendations for the Extraction, Analysis, and Presentation of Results in Scoping Reviews,” JBI Evidence Synthesis 21, no. 3 (2023): 520–532. 36081365 10.11124/JBIES-22-00123

[10] AGREE Enterprise , “The AGREE Next Steps Consortium,” (2017). https://www.agreetrust.org/wp-content/uploads/2017/12/AGREE-II-Users-Manual-and-23-item-Instrument-2009-Update-2017.pdf.

[11] C. Maaser , A. Sturm , S. R. Vavricka , et al., “ECCO‐ESGAR Guideline for Diagnostic Assessment in IBD Part 1: Initial Diagnosis, Monitoring of Known IBD, Detection of Complications,” Journal of Crohn's and Colitis 13, no. 2 (2019): 144–164K. 10.1093/ecco-jcc/jjy113 30137275

[12] T. Kucharzik , A. U. Dignass , R. Atreya , et al., “(2019). Updated S3‐Guideline Ulcerative Colitis. German Society for Digestive and Metabolic Diseases (DGVS),” Journal of Gastroenterology 57, no. 02 (2029): 162–241. 10.1055/a-0824-0861 30654406

[13] S. Kedia , R. Sharma , G. Makharia , et al., “Indian Guidelines on Imaging of the Small Intestine in Crohn's Disease: A Joint Indian Society of Gastroenterology and Indian Radiology and Imaging Association Consensus Statement,” Indian Journal of Radiology and Imaging 29, no. 2 (2019): 111–132. 31367083 10.4103/ijri.IJRI_153_18 PMC6639863

[14] D. T. Rubin , A. N. Ananthakrishnan , C. A. Siegel , B. G. Sauer , and M. D. Long , “ACG Clinical Guideline: Ulcerative Colitis in Adults,” American Journal of Gastroenterology 114, no. 3 (2019): 384–413. 30840605 10.14309/ajg.0000000000000152

[15] D. H. Kim , K. J. Chang , K. J. Fowler , et al., “ACR Appropriateness Criteria® Crohn Disease,” supplement, Journal of the American College of Radiology 17, no. 5 (2020): S81–S99. 32370980 10.1016/j.jacr.2020.01.030

[16] M. Alkhatry , A. Al‐Rifai , V. Annese , et al., “First United Arab Emirates Consensus on Diagnosis and Management of Inflammatory Bowel Diseases: A 2020 Delphi Consensus,” World Journal of Gastroenterology 26, no. 43 (2020): 6710–6769. 33268959 10.3748/wjg.v26.i43.6710 PMC7684461

[17] H. Nakase , M. Uchino , S. Shinzaki , et al., “Evidence‐Based Clinical Practice Guidelines for Inflammatory Bowel Disease 2020,” Journal of Gastroenterology 56, no. 6 (2021): 489–526. 33885977 10.1007/s00535-021-01784-1 PMC8137635

[18] M. Łodyga , P. Eder , M. Gawron‐Kiszka , et al., “Guidelines for the Management of Patients With Crohn's Disease. Recommendations of the Polish Society of Gastroenterology and the Polish National Consultant in Gastroenterology,” Gastroenterology Review 16, no. 4 (2021): 257–296. 34976235 10.5114/pg.2021.110914 PMC8690943

[19] M. H. Mosli , H. Y. Almudaiheem , T. AlAmeel , et al., “Saudi Arabia Consensus Guidance for the Diagnosis and Management of Adults With Inflammatory Bowel Disease,” Saudi Journal of Gastroenterology 29, no. 1 (2022): 1–35. 10.4103/sjg.sjg_277_22 PMC10540981 36412460

[20] P. Eder , M. Łodyga , M. Gawron‐Kiszka , et al., “Guidelines for the Management of Ulcerative Colitis. Recommendations of the Polish Society of Gastroenterology and the Polish National Consultant in Gastroenterology,” Gastroenterology Review 18, no. 1 (2023): 1–42. 37007752 10.5114/pg.2023.125882 PMC10050986

[21] Y. A. Shelygin , V. T. Ivashkin , S. I. Achkasov , et al., “Clinical Guidelines Crohn's Disease (К50), Adults,” Coloproctology 22, no. 3 (2023): 10–49.

